# A Health Monitoring Model for Circulation Water Pumps in a Nuclear Power Plant Based on Graph Neural Network Observer

**DOI:** 10.3390/s24144486

**Published:** 2024-07-11

**Authors:** Jianyong Gao, Liyi Ma, Chen Qing, Tingdi Zhao, Zhipeng Wang, Jie Geng, Ying Li

**Affiliations:** 1 National Engineering Research Center for Nuclear Power Plant Safety & Reliability, Suzhou 215004, China; gao19892008@gmail.com (J.G.); qingchen@cgnpc.com.cn (C.Q.); 2 Suzhou Nuclear Power Research Institute Co., Ltd., Suzhou 215004, China; 3 School of Traffic and Transportation, Beijing Jiaotong University, Beijing 100044, China; 23111294@bjtu.edu.cn (L.M.); zpwang@bjtu.edu.cn (Z.W.); 4 School of Reliability and Systems Engineering, Beijing University of Aeronautics and Astronautics, Beijing 100191, China; ztd@buaa.edu.cn (T.Z.); gengjie@buaa.edu.cn (J.G.)

**Keywords:** health monitoring model, CRF pump, graph self-learning, fault observer

## Abstract

The health monitoring of CRF (circulation water) pumps is essential for prognostics and management in nuclear power plants. However, the operational status of CRF pumps can vary due to environmental factors and human intervention, and the interrelationships between monitoring parameters are often complex. Consequently, the existing methods face challenges in effectively assessing the health status of CRF pumps. In this study, we propose a health monitoring model for CRF pumps utilizing a meta graph transformer (MGT) observer. Initially, the meta graph transformer, a temporal–spatial graph learning model, is employed to predict trends across the various monitoring parameters of the CRF pump. Subsequently, a fault observer is constructed to generate early warnings of potential faults. The proposed model was validated using real data from CRF pumps in a nuclear power plant. The results demonstrate that the average Mean Absolute Percentage Error (MAPE), Mean Absolute Error (MAE), and Root Mean Square Error (RMSE) of normal predictions were reduced to 1.2385, 0.5614, and 2.6554, respectively. These findings indicate that our model achieves higher prediction accuracy compared to the existing methods and can provide fault warnings at least one week in advance.

## 1. Introduction

A stable and reliable operation of the CRF pump in a nuclear power plant is essential for ensuring nuclear power safety. If the CRF pump breaks down, the generator unit must be downrated to match the cooling decline in the unit, resulting in an impact on the capacity output of the nuclear power plant [1,2]. To ensure the normal operation of the CRF pump under complex and variable working conditions, a large number of sensor sources are used to monitor the parameters of the CRF pump, providing data support for the subsequent fault diagnosis and health monitoring of the CRF pump.

Concerning this background, recent advancements have focused on sophisticated methods to diagnose faults and predict the remaining useful life (RUL) of CRF pumps in nuclear power plants or other mechanical facilities utilizing the latest advancements in data-driven approaches, signal processing, and machine learning [3,4,5,6,7,8,9,10,11,12,13,14,15,16]. Domain adaptation has been effectively used to improve cross-domain bearing fault diagnosis in CRF pumps [2]. Additionally, an optimized MOMEDA approach has enhanced fault detection in planetary bearings [17]. To address the challenge of data imbalance in health state identification, a technique utilizing multi-virtual vibration source fusion was introduced, which has improved the reliability of monitoring systems [18]. Probabilistic approaches have also been applied successfully. R-vine copula has enabled robust monitoring under uncertain conditions [19], while the Bayesian inference has provided precise uncertainty quantification, crucial for the reliability of CRF pumps [20]. Hybrid models have shown great promise, such as an interactive hybrid model that predicts the remaining useful life of CRF pump bearings while incorporating uncertainty quantification for enhanced reliability [21]. Another study advanced prognosis in CRF pumps using a novel health indicator combined with a nonlinear Wiener process [22]. Innovative machine learning techniques have also played a pivotal role. An adaptive fault diagnosis methodology based on NSGAII-CNN combined evolutionary algorithms with deep learning to improve fault detection in CRF pumps [23]. Furthermore, an open set recognition framework using convolutional prototype learning networks addressed the challenge of unknown fault categories [11]. Collectively, these approaches have significantly enhanced the robustness, accuracy, and efficiency of health monitoring systems for CRF pumps, ensuring their reliable operation in nuclear power plants [24]. In the field of other mechanical facilities, a method for diagnosing intermittent faults in electronic circuits by enhancing and locating local features has improved accuracy and reliability [25]. An ultrafast structural damage identification framework using an optimized extreme learning machine has provided rapid and reliable detection [26]. Deep learning and transfer learning have also been extensively explored. An AI-based framework for rotating machinery leverages these methods to achieve high accuracy and adaptability [27]. Multi-sensor data integration has enhanced the robustness and accuracy of nuclear power plant monitoring systems [28]. AI applications in machine condition monitoring and fault diagnosis have shown substantial potential [29]. Advanced machine learning techniques, such as self-adaptive GNNs, enhance predictive capabilities in health monitoring systems. For example, a deep dynamic high-order GNN for the wear fault diagnosis of hydrodynamic mechanical seals captures dynamic behaviors and improves fault diagnosis [30]. Integrating data-augmentation techniques with GNNs further enhances fault diagnosis performance in rotating machinery [31]. Multi-sensor data fusion techniques are crucial for improving fault detection accuracy and reliability. A novel fusion technique integrates data from various sensors [32]. Pruning quantized unsupervised meta-learning frameworks have been effective in industrial and semiconductor anomaly detection [33].

The existing methods can realize the health monitoring of traditional mechanical facilities, but they cannot cope with the characteristics of monitoring data from the CRF pumps in nuclear plants, nor can they cope with the impact of working conditions or environmental changes on the monitoring model [8]. Specifically, the existing methods often overlook the unique characteristics of monitoring data from CRF pumps. These pumps are complex systems with multiple components, including motors, pump bodies, and gearboxes, requiring the monitoring of numerous variables. Most studies focus on only one or a few variables. However, the operational status of CRF pumps frequently changes due to environmental factors and human interventions, leading to variations in many monitoring variables and complex correlations among them. Consequently, considering only a limited set of variables is insufficient for accurately predicting the health status of CRF pumps under varying operational conditions [34,35,36,37,38,39,40].

To address these challenges, advanced methodologies named graph neural networks (GNNs) have emerged as powerful tools. Among these, MGT stands out for its performance and ability to process multi-source spatiotemporal data. In recent years, MGT has been primarily applied to spatiotemporal data processing in areas such as traffic prediction [41,42,43,44,45,46], social network analysis [47,48,49,50,51,52], bioinformatics [53,54,55,56,57,58], and medical data analysis [59,60,61,62,63]. Although specific case studies in fault diagnosis and health monitoring are limited, the core technologies and methodologies of MGT have significant potential in these areas. The framework of MGT is designed to handle complex, interconnected systems like CRF pumps by modeling the intricate relationships and dependencies between various components and their operational states. It excels in capturing spatiotemporal patterns within the monitoring data, making it particularly effective for systems subject to dynamic changes in operational conditions and environmental factors. By leveraging the capabilities of MGT, it becomes possible to integrate multiple variables and adapt to fluctuating conditions, thereby enhancing the accuracy and robustness of health prediction. This approach provides a comprehensive and adaptive solution to the limitations of traditional methods, ensuring the reliable monitoring and maintenance of CRF pump systems. Furthermore, a fault observer based on MGT is also proposed in this paper; compared to traditional fault observers, the MGT-based fault observer can capture higher-order relationships and dependencies within the data, which conventional observers may overlook. This leads to a more comprehensive understanding of complex systems and interactions. Secondly, meta graph transformers are more adaptable and scalable to large and diverse datasets, allowing for more accurate and robust predictions [64,65,66,67,68,69].

In view of the above situation, this paper focuses on advanced health monitoring for CRF pumps using MGT. By leveraging correlations between various monitoring data, a graph constructed from this data serves as the input for MGT. This innovative approach predicts trends in the monitoring data, effectively addressing challenges posed by the diverse and voluminous data of CRF pumps, enabling the precise identification of changing working conditions. Additionally, a fault observer based on MGT is developed to forecast potential faults and provide early warnings. This significantly enhances the prediction accuracy of CRF pump parameters, thereby facilitating the robust health monitoring of the system. The proposed method demonstrates substantial engineering value and significance by improving maintenance efficiency and reducing downtime.

Therefore, the main contributions and novelty of this paper are as follows:

1. The concept of graph self-learning is introduced, utilizing a graph self-learning layer to adaptively extract relationships between the multivariate monitoring data of the CRF pump. This approach ensures that the graph neural network is no longer influenced by the diversity of predefined graph structures when predicting the node characteristics within the graph structure.
2. The graph structure can be continuously updated during the training process, enabling the system to better handle multiple working conditions and monitor the variable changes in the CRF pumps in nuclear plants over time.
3. Combined with the graph self-learning neural network model MGT, a fault observation system for CRF pumps is constructed. By training on normal data to predict conditions during the fault observation phase, the deviations of the actual values from the predicted values within a certain range indicate potential issues that require attention. This system can monitor the fault conditions of CRF pumps and provide early warnings.

This paper is organized as follows: Section 1 provides an overview of the research object. Section 2 details the proposed health monitoring model with graph self-learning. In Section 3, real data from the CRF pump is used for verification experiments, and the results are discussed. Finally, Section 4 concludes the paper.

## 2. Methodology

This section presents the proposed health monitoring model for the CRF pump. First, multi-source sensor data are collected from the CRF pump. A graph structure is then constructed based on a quantitative analysis of the Wasserstein distance, and this graph structure is used as input for the MGT model to predict the health status of the CRF pump. Additionally, a CRF pump fault observer based on MGT is designed. The estimated output of the MGT fault observer is compared with the actual monitoring data to obtain the residual error. When the residual error exceeds a predefined threshold, it indicates an abnormal trend in the monitoring parameters of the CRF pump, triggering early warning information and enabling early fault detection.

**Figure 1:**
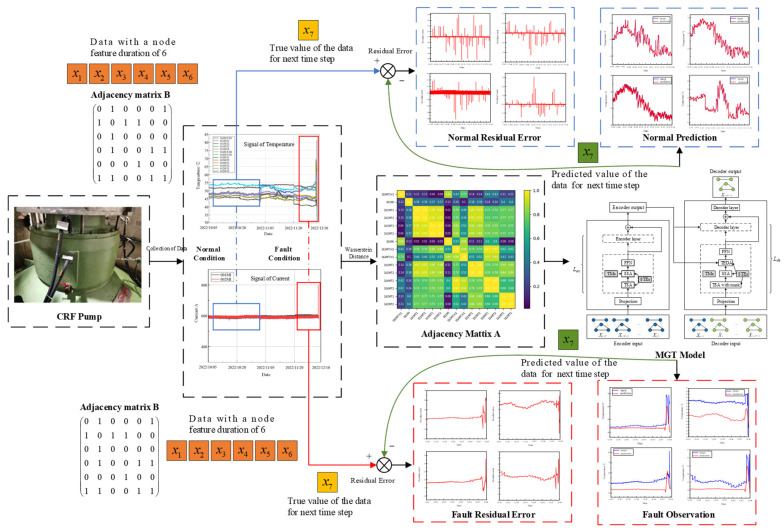
Structure of proposed health monitoring model.

The structure of the proposed MGT observer health monitoring model is shown in Figure 1. This model leverages advanced graph learning techniques to dynamically update the graph structure during operation, enhancing its ability to adapt to different operating conditions. The continuous update mechanism allows for real-time monitoring and timely interventions, thus reducing the risk of unexpected failures. Moreover, the integration of diverse data sources ensures the comprehensive coverage of potential fault indicators, making the health monitoring system more robust and reliable. This comprehensive approach ensures a robust monitoring system, enhancing the reliability and operational efficiency of CRF pumps.

### 2.1. Quantitative Analysis of Wasserstein Distance

Since the data collected from the 15 sensor groups used in this study are all derived from the CRF pump system, there is an inherent connection in the physical structure. The changes in its operation are expected to exhibit certain regularities and systematic patterns. The correlation analysis of the 15 sets of sensor data is performed using the Wasserstein distance method to verify the data correlation, providing a theoretical basis for the subsequent construction of the CRF pump graph data.

The Wasserstein distance measures the similarity between two distributions and can be understood as the minimum amount of movement required to transform one distribution into another. Therefore, the Wasserstein distance between the monitoring data can be regarded as the weight of the edges between the data points. Given two probability distributions, the Wasserstein distance between them is defined as follows:

(1) $$l(u,v)=\underset{\pi \in \Gamma (u,v)}{\inf}\int_{R\times R}\left|x-y\right|d\pi (x,y)$$

where Γ(u,v) is the probability distribution on R×R.

Considering the integrity and systematic nature of the monitoring data, it is evident that different monitoring data exhibit varying degrees of similarity. The Wasserstein distance between the monitoring data is calculated to measure this similarity, resulting in a similarity matrix. Due to significant differences in the dimensions and distributions of the data, the calculated Wasserstein distance can span a wide range. To mitigate the influence of these differences on subsequent data analysis, the smaller the Wasserstein distance, the greater the similarity between the data. Therefore, an exponential function is used to normalize the similarity matrix. The normalized similarity is given by the following:

(2) s=exp(−l(u,v))

Given the large size of the similarity matrix and the concentration of many values between 0 and 0.1, it is necessary to sparsify the matrix to reduce computational complexity while retaining as much information as possible. By setting a threshold of 0.1, the similarity matrix can be updated as follows:

(3) $$s=\left\{ \begin{array}{l} s,s\geq 0.1\\ 0,s<0.1 \end{array}\right.$$

### 2.2. Meta Graph Transformer

**Figure 2:**
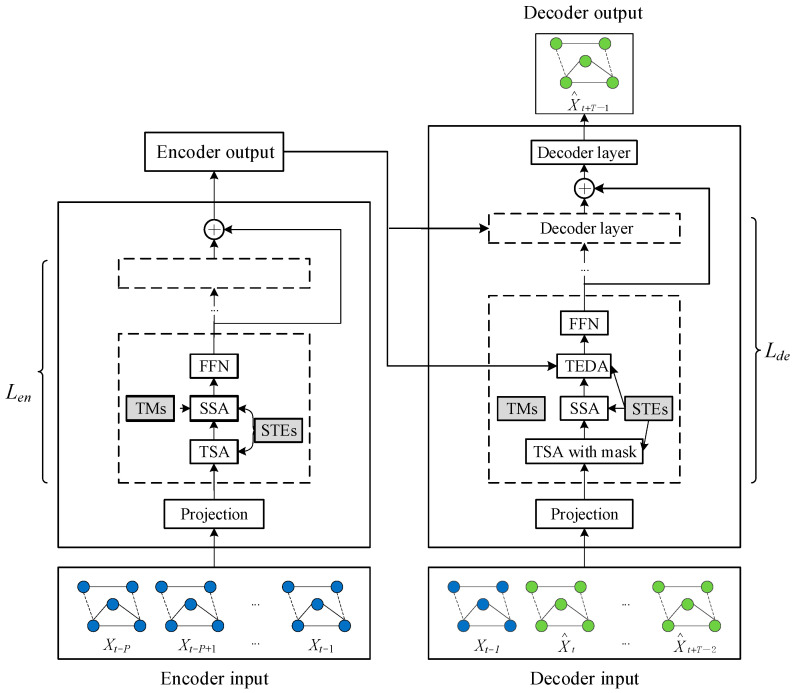
Framework of MGT.

The main concept of the MGT model is to establish a spatial–temporal heterogeneity attention (STHA) layer guided by external spatial–temporal attributes, enabling the learning of complex spatial–temporal correlations between data. The overall framework of the MGT is illustrated in Figure 2.

The model features an encoder–decoder structure, with both encoders and decoders composed of multiple identical layers. To enhance the model’s learning capability, skip connections are utilized. Within the sub-layers, an attention mechanism is employed to uncover implicit patterns in the spatial and temporal data. The three attention layers are named the temporal self-attention layer (TSA), the spatial self-attention layer (SSA), and the temporal encoder–decoder attention layer (TEDA). Each attention layer has a multi-head structure and uses spatial–temporal embeddings learned from external spatial–temporal attributes to achieve spatial–temporal heterogeneity perception. Additionally, the spatial self-attention layer comprehensively integrates information from multiple graphs to explore complex and diverse spatial correlations.

In the meta graph transformer, the input of the encoder is the historical monitoring data $X\in {\mathbb{R}}^{P\times N\times C}$. This historical monitoring data is mapped to $X^{\left(0\right)}\in {\mathbb{R}}^{P\times N\times d_{\mathrm{model}}}$ using the ReLU function and a linear transformation, where $d_{\mathrm{model}}$ represents the input dimension of the model. The projected feature $X^{\left(0\right)}$ is fed into $L_{en}$ identical encoder layers with skip connections. Assuming that the output of the *l* encoder layer is $X^{\left(l\right)}$, the output of the encoder is shown as follows:

(4) $$X_{en}=\sum_{l=1}^{L_{en}}X^{\left(l\right)}\in {\mathbb{R}}^{P\times N\times d_{\mathrm{model}}}$$

where *P* is the number of time steps in the historical data; *N* is the number of monitoring points; *C* is the number of features at each monitoring point; $d_{\mathrm{model}}$ is the dimension of the model input.

Based on $X_{en}$, the decoder can predict future monitoring data trends. It uses previous predictions as inputs and feeds them into $L_{en}$ identical decoder layers with skip connections. Finally, a linear transformation projects the features into $d_{\mathrm{model}}$, which represents the future values of the monitoring data at the next time step. This step is repeated for *H* times to forecast the future states of the monitoring data.

#### 2.2.1. Embedding of Spatial–Temporal

The purpose of spatial–temporal embedding is to map complex spatial–temporal data into a lower-dimensional vector space, facilitating the application of various deep learning methods for modeling and analysis. The original author of MGT considers traffic data as a typical example of spatial–temporal data and transforms it into a fixed-length mathematical vector using spatial–temporal embedding. This transformation represents the external spatial–temporal attributes contained within the data.

#### 2.2.2. Construction of Adjacency Matrix

The adjacency matrix stores the correlations between nodes in a graph structure, and its construction methods are complex and varied. To maximize the extraction of implicit information between nodes and enhance the stability and reliability of the graph structure, various methods can be employed to construct the adjacency matrix. In the MGT model, the adjacency matrix is constructed by determining the edge weights based on the actual distance between nodes in the traffic network and the OD information reflected by the traffic flow data. When applying the MGT model to the health monitoring of CRF pumps, it is essential to select an appropriate method for constructing the adjacency matrix according to the characteristics of the monitoring data.

#### 2.2.3. Encoder

The encoder employs a skip connection to stack an input projection layer and an identical encoder layer. Each encoder layer comprises three components: a temporal self-attention layer, a spatial self-attention layer, and a feedforward network layer (FFN). Both the temporal and spatial self-attention layers utilize a multi-head structure, responsible for extracting temporal and spatial correlations, respectively. These layers can perform spatial–temporal heterogeneity perceptual attention operations, making the learning process more adaptable to specific spatial–temporal conditions. By using multiple graphs simultaneously, the spatial self-attention layer can also capture spatial correlations from different perspectives. Connecting these three components sequentially enables the extraction of spatial–temporal characteristic information.

Given that the traffic state exhibits dynamic changes at different times and locations, the time multi-head self-attention layer cannot account for this factor. Therefore, the model proposes an STE-guided multi-head time self-attention mechanism. The main idea is to replace shared parameters with corresponding STE functions, enabling the time self-attention layer to perform spatial–temporal heterogeneity perception self-attention operations. Specifically, this involves creating a multi-layer perceptron with a hidden layer for each head, as shown in Formula (5). Additionally, residual connections and layer normalization can be used to optimize the network.

(5) $$MLP(x)=W_{2}^{mlp}ReLU(W_{1}^{mlp}x+b_{1}^{mlp})+b_{2}^{mlp}$$

Simultaneously, an STE-guided multi-head multi-graph spatial self-attention mechanism is designed to extract spatial features. The use of multiple graphs helps to distinguish different spatial locations, focus on nodes that are more closely related to the central node, and capture the various types of relationships between nodes.

The feedforward network in the encoder is a position transformation layer, and the parameters of each space–time point are shared. Given a feature vector $x\in R_{\mathrm{model}}^{d}$, the expression of the feedforward network is shown as the following formula.

(6) $$y=LayerNorm(x+W_{2}^{ffn}ReLU(W_{1}^{ffn}x+b_{1}^{ffn})+b_{2}^{ffn})$$

#### 2.2.4. Decoder

Each decoder layer consists of four components, namely, the temporal self-attention layer with masks, the spatial self-attention layer, the temporal encoder–decoder attention layer, and the feedforward network layer. The behavior of the time self-attention layer is masked to prevent the model from using future information during training. The mask is usually designed as a matrix of T×T, with the diagonal element of −∞ and the other elements of 0. The time encoder–decoder attention layer is used to connect the output of the encoder and each decoder layer, and can learn adaptive features from historical data.

### 2.3. MGT Fault Observer

**Figure 3:**
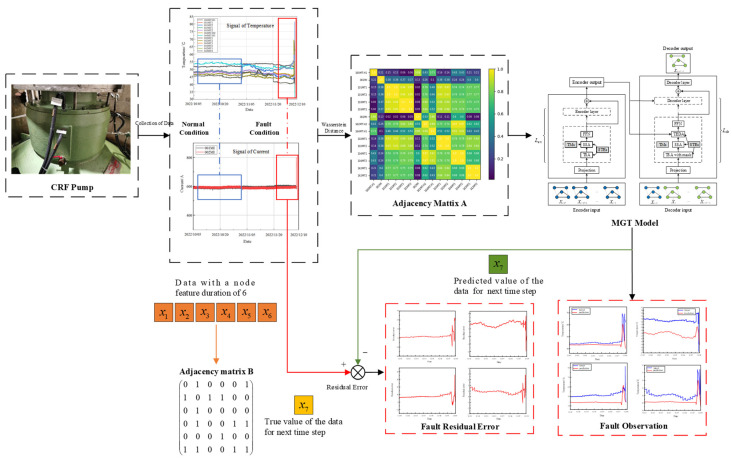
Structure of MGT fault observer.

Combining the traditional observer with the MGT model, the structure diagram of the fault observer based on MGT can be obtained as shown in Figure 3. In the figure, the health monitoring and fault diagnosis process for a CRF pump using the MGT model is depicted. Data collection occurs at the CRF pump (top left), represented by node features (*x*_1_, *x*_2_, *x*_3_, *x*_4_, *x*_5_, and *x*_6_), and is used to construct adjacency matrix B. These data are processed to identify normal and fault conditions, illustrated by the graphs of temperature and current signals. The adjacency matrix A (middle right) captures relationships among sensor readings, aiding in system behavior analysis. The MGT model (right) consists of encoder and decoder components. The encoder processes input data, and the decoder predicts the next time step value (*x*_7_), which is compared to the true value to calculate the residual error. The residual error graphs (bottom right) distinguish between normal and fault conditions, aiding fault observation. This comprehensive layout demonstrates how the MGT model uses spatiotemporal data to improve the accuracy and robustness of health predictions for CRF pumps.

When training the MGT fault observer, two types of data are utilized. The first type is the adjacency matrix of the graph structure of the monitoring data at a time step of 6. The second type is the data for the next time step following the node feature data of the graph structure of the monitoring data. A large number of samples are used to train the MGT fault observer to determine the parameters of the MGT model. The difference between the actual value of the data at the next time step and the predicted value by the MGT fault observer is shown as follows:

(7) $$\varepsilon (k)=y_{r}(k)-{\hat{y}}_{r}(k)$$

When the MGT fault observer is trained using data samples from the normal state, the parameters of the MGT model are determined and remain unchanged. These parameters encapsulate various state information during normal operation. Under normal conditions, the difference between the estimated output and the actual output of the MGT fault observer is small and generally fluctuates around zero.

In fault prediction, residuals can be used as important reference information. A reasonable fixed threshold is determined by using expert knowledge and other experience. If the residual error exceeds the threshold, it indicates a malfunction in the CRF pump; conversely, if the residual error is below the threshold, it suggests that the CRF pump is operating normally.

## 3. Experimental Results and Analysis

### 3.1. Experimental Setup

**Figure 4:**
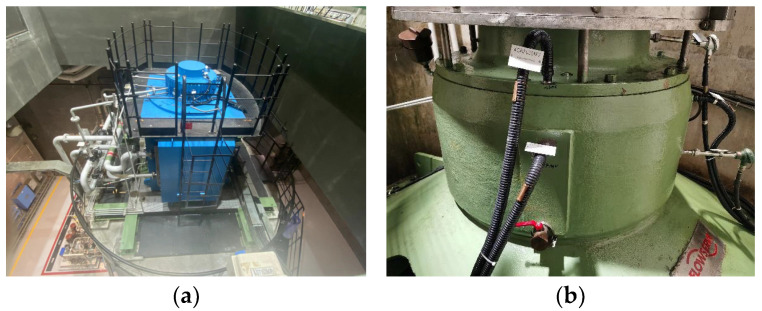
CRF pump in a nuclear plant: (**a**) sea water cooling system; (**b**) CRF pump.

To demonstrate the proposed health monitoring model, real data obtained from the CRF pump in a nuclear plant, as shown in Figure 4, are used.

**Figure 5:**
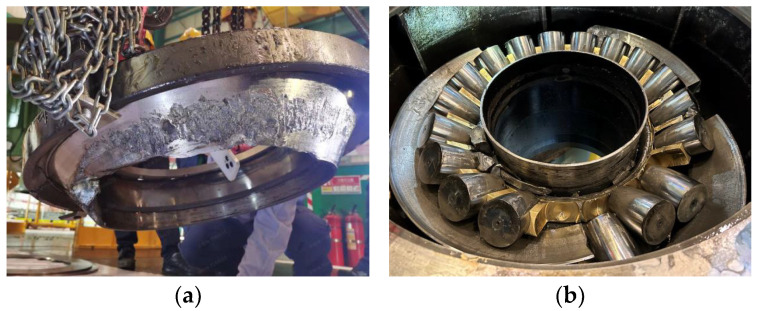
Fault of CRF pump: (**a**) fault section a of thrust bearing; (**b**) fault section b of thrust bearing.

On 7 December, the CRF pump of the nuclear power plant was damaged due to a thrust bearing fault, as shown in Figure 5. This resulted in the entire system being unable to operate and shutting down for maintenance.

#### 3.1.1. Datasets

**Figure 6:**
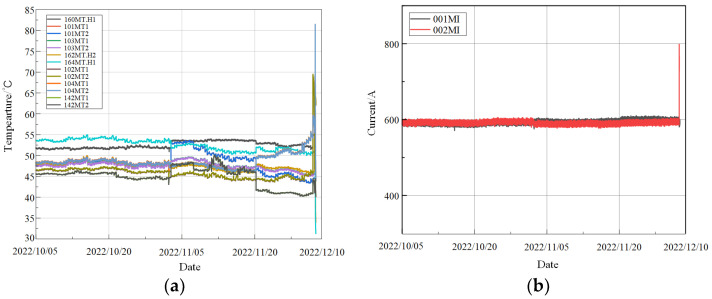
Collected data from sensors: (**a**) data of temperature signals; (**b**) data of current signals.

To analyze and address the fault situation and prevent recurrence, data from 15 sensors arranged on the CRF pump were collected, as shown in Figure 6. Table 1 provides a comprehensive overview of the multi-source sensor data collected from the CRF pump system. It lists the measuring points, respective locations, and units of measurement. Sensors were installed at critical points such as the output thrust bearing of the gearbox, the current of the motor, and various bearings of the motor and pump. These sensors monitored essential parameters such as amperes (A) for current and degrees Celsius (°C) for temperature, crucial for accurately assessing the pump’s health and operational status. These multi-source data are integral for constructing the input graph for the MGT model, enabling robust health monitoring and fault detection for the CRF pump. Each sensor collects data every six seconds. During the normal operation of the equipment, 460,482 data points were collected, and 85,000 data points were collected on the day of the failure and the preceding period. This resulted in a matrix of the original data used in the experiment. The data types were primarily integers and floating-point numbers, with a small number of Boolean values. Since each data group represents different monitoring parameters, they may have different units.

To eliminate the influence of different dimensions, 15 sets of monitoring data were standardized using the Z-score method. The data were then divided as shown in Table 2. The analysis period spans from 5 October to 8 December, during which the environmental temperature at the plant location remained relatively stable and was considered negligible. The data collected from 5 October to 16 November, totaling 460,482 entries, were used for quantitative correlation analysis, as the CRF pump was operating normally during this period. The training set comprised 222,000 data points collected from 5 October to 19 October, a period when the CRF pump was functioning normally and was distant from the failure date, and thus was unaffected. The data from 20 October to 25 October, totaling 70,000 entries, were used as a validation set to buffer and ensure no overlap between the training and test sets. The normal operation test for the CRF pump employed 140,082 data points collected from 5 November to 16 November to verify the model’s predictive performance under normal operating conditions. To meet the actual engineering needs of the plant, it is necessary to monitor the equipment’s operation before a fault to provide early fault warnings. Therefore, 85,000 data points from 1 December to 8 December were used as the fault test set to verify the reliability and effectiveness of the proposed method.

**Figure 7:**
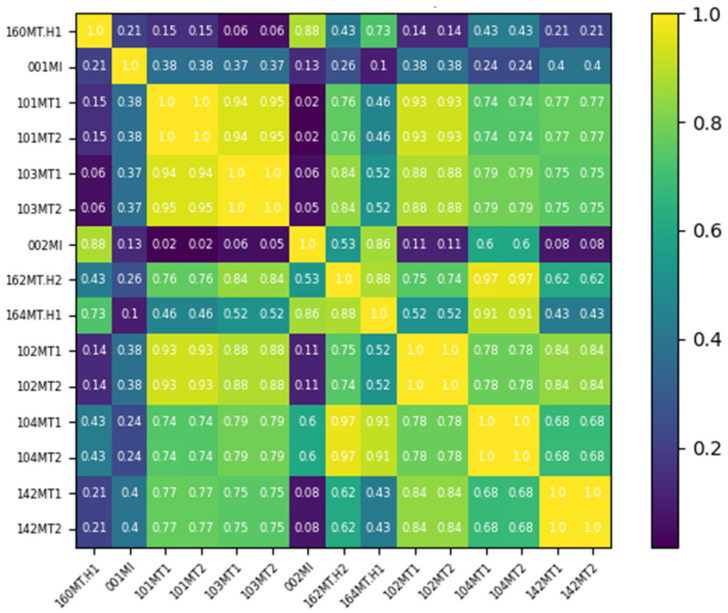
Wasserstein distance of adjacency matrix.

The Wasserstein distance is used to quantitatively construct the adjacency matrix for the graph structure of the monitoring data, as shown in Figure 7. This matrix serves as the input for the CRF pump health monitoring model based on the MGT observer.

#### 3.1.2. MGT Parameter Settings

The encoder in the MGT model uses six encoding layers, and the decoder uses six decoding layers. To achieve better training results, a dropout layer with a rate of 0.3 is added before the residual connection. The training batch size is set to two. The meta graph transformer model employed in this study uses the Adam optimizer, which is suitable for large datasets. The initial learning rate is 0.001, and the weight decay is 0.0002. The model was trained for 300 epochs, and after 150 epochs, the learning rate decreased by 0.1.

#### 3.1.3. Evaluation Metrics Settings

RMSE, MAE, and MAPE are used to evaluate the prediction performance of the models on each test. RMSE is a common measure of the error between predicted and observed values, which is denoted as follows:

(8) $$\mathrm{RMSE}=\sqrt{\frac{1}{n}\sum_{i=1}^{n}{\left(predicted_{i}-observed_{i}\right)}^{2}}$$

MAE is denoted as follows:

(9) $$\mathrm{MAE}=\frac{1}{n}\sum_{i=1}^{n}\left|predicted_{i}-observed_{i}\right|$$

MAPE is less sensitive to outliers compared to RMSE. It is calculated as follows:

(10) $$\mathrm{MAPE}=\frac{100\%}{n}\sum_{i=1}^{n}\left|\frac{predicted_{i}-observed_{i}}{observed_{i}}\right|$$

### 3.2. Evaluation of the Proposed Health Monitoring Model

The sensor data from 102 MT_1_, 103 MT_1_, 104 MT_1_, and 142 MT_1_ were utilized for analysis based on the testability analysis and engineering experience of the nuclear plant. These specific sensors were selected due to their critical role in monitoring key parameters within the nuclear plant’s operational framework. The comprehensive analysis of these sensor readings provides valuable insights into the plant’s performance and reliability. This approach ensures that the data used for the health monitoring and fault diagnosis processes is both relevant and reliable, ultimately enhancing the accuracy and robustness of the predictive maintenance models employed in the nuclear plant.

#### 3.2.1. Prediction of Normal Condition

**Figure 8:**
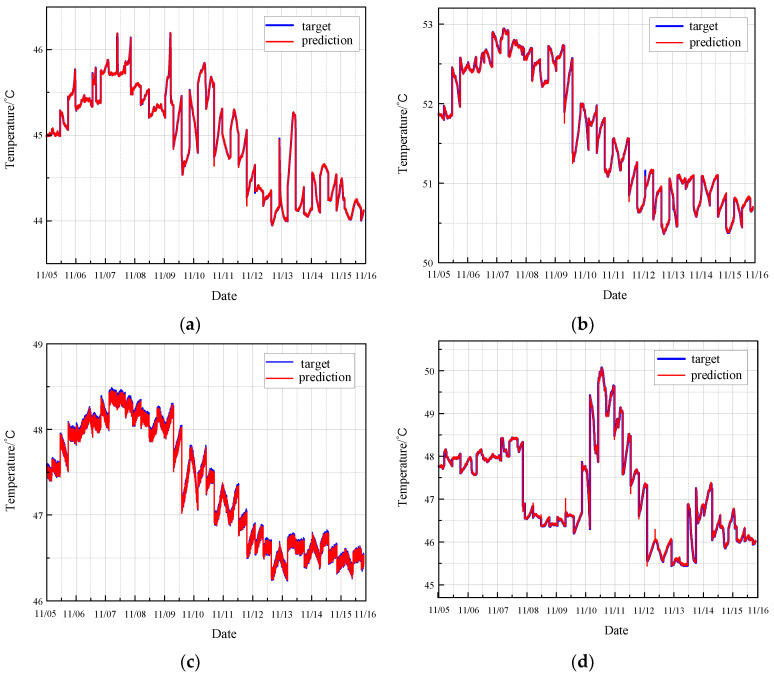
Prediction of normal condition based on MGT: (**a**) results of sensor 102 MT_1_; (**b**) results of sensor 103 MT_1_; (**c**) results of sensor 104 MT_1_; (**d**) results of sensor 142 MT_1_.

Firstly, using the normal data from the four sensors on the CRF pump, the MGT fault observer predicts the monitoring parameters for the next time step following a sequence of six steps. The comparison between the predicted value and the actual value of the monitoring data of the CRF pump is shown in Figure 8.

The results demonstrate that the MGT algorithm effectively predicts the monitoring parameters of the CRF pump. It excels in accurately tracking the trends and variations in these parameters over time. Moreover, the MGT algorithm is particularly adept at identifying significant changes in the monitoring data, even those with substantial intensity. This capability underscores the algorithm’s robustness and reliability in the real-time health monitoring and fault diagnosis of CRF pumps. The enhanced tracking and identification abilities of the MGT algorithm provide critical insights into the pump’s operational status, thereby facilitating timely maintenance and ensuring the overall efficiency and safety of the nuclear plant operations.

**Figure 9:**
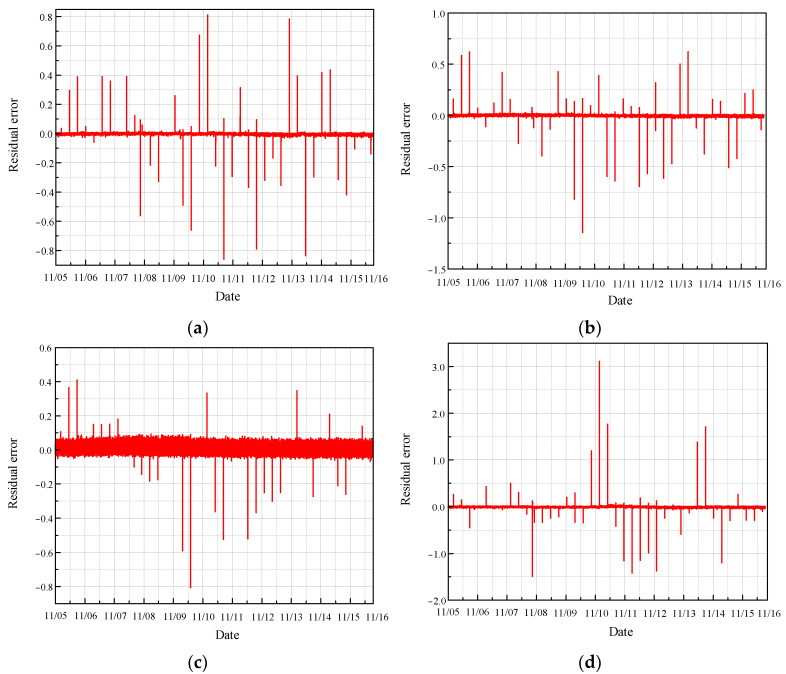
Residual error of normal condition based on MGT: (**a**) results of sensor 102 MT_1_; (**b**) results of sensor 103 MT_1_; (**c**) results of sensor 104 MT_1_; (**d**) results of sensor 142 MT_1_.

The real values of the monitoring parameters were then compared with the predicted values, and the residuals, representing the difference between these real and predicted values, were calculated. The length of the residual data is 140,082, as illustrated in Figure 9.

As illustrated in the figure, most of the residual values fluctuate around zero, indicating that the temperature parameters from the normal sections of the four sensors are relatively stable and within the safe range. This stability suggests that the CRF pump is operating under normal conditions without significant deviations or anomalies in the monitored temperature parameters. The minimal fluctuations around zero demonstrate the effectiveness of the MGT model in accurately predicting the normal operational state of the CRF pump. This reliable performance is crucial for maintaining the safety and efficiency of the system, as it ensures that any significant deviations from the norm can be promptly detected and addressed.

#### 3.2.2. Contrast of Other Methods

To verify the superiority of the CRF pump health monitoring model based on the MGT proposed in this article, several classical univariate time series prediction methods are used for comparative experiments. These methods include long short-term memory (LSTM) networks, convolutional neural networks (CNNs), Deep AR, and temporal convolutional networks (TCNs). The training set, validation set, and test set used for the other prediction models are the same as those for the MGT model, as shown in Table 2. The experimental results are compared with the results of the MGT-based CRF pump health monitoring model to evaluate performance.

(1) Long short-term memory network

**Figure 10:**
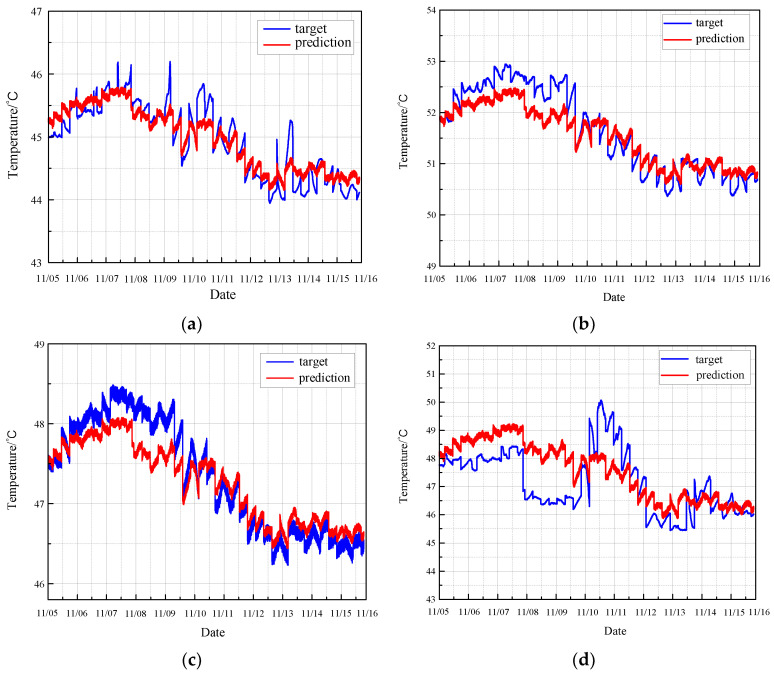
Prediction of normal condition based on LSTM: (**a**) results of sensor 102 MT_1_; (**b**) results of sensor 103 MT_1_; (**c**) results of sensor 104 MT_1_; (**d**) results of sensor 142 MT_1_.

The long short-term memory (LSTM) network is employed to predict the monitoring data of the CRF pump in its normal state. The LSTM model predicts the data of the seventh time step using the data from the preceding six continuous time steps. The experimental results for the normal monitoring data of the CRF pump are presented in Figure 10.

**Figure 11:**
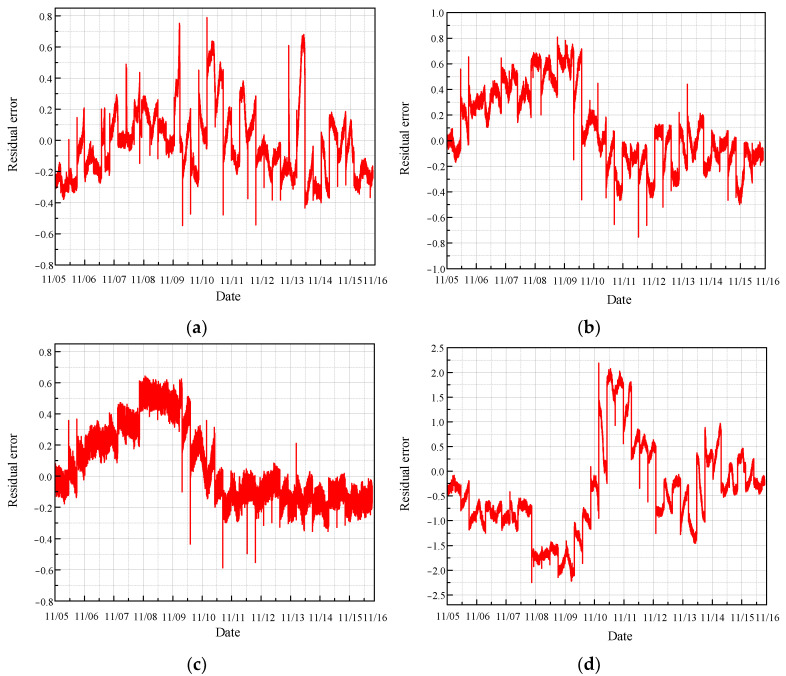
Residual error of normal prediction based on LSTM: (**a**) results of sensor 102 MT_1_; (**b**) results of sensor 103 MT_1_; (**c**) results of sensor 104 MT_1_; (**d**) results of sensor 142 MT_1_.

The residual error between the predicted values and the true values for normal conditions, as determined by the LSTM model, is illustrated in Figure 11.

Based on the LSTM prediction results, it is evident that the LSTM method performs poorly in predicting the data. Although the general trend is followed, the prediction accuracy is suboptimal. Additionally, the residuals observed show large and irregular fluctuations, further demonstrating the ineffectiveness of the LSTM method in this paper.

(2) Convolutional neural network

**Figure 12:**
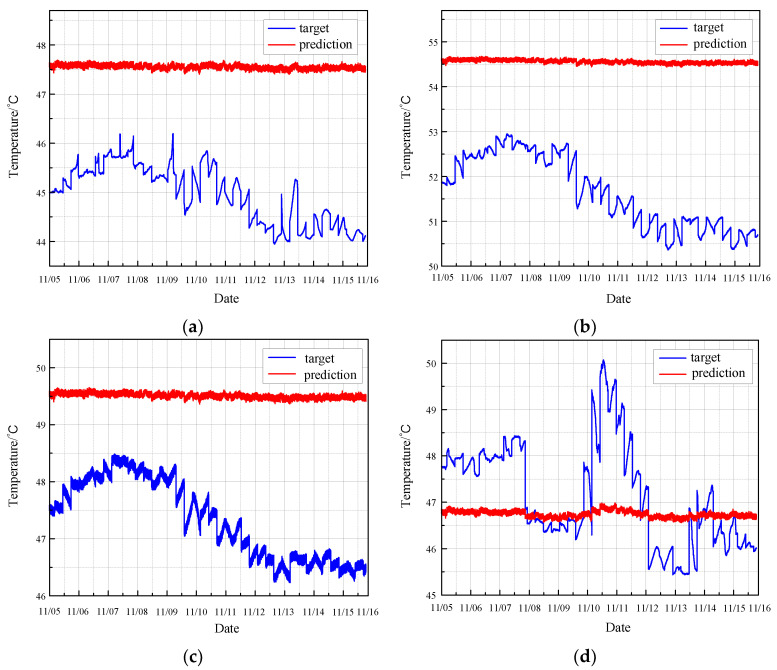
Prediction of normal condition based on CNN: (**a**) results of sensor 102 MT_1_; (**b**) results of sensor 103 MT_1_; (**c**) results of sensor 104 MT_1_; (**d**) results of sensor 142 MT_1_.

Following the same rule, a convolutional neural network (CNN) is employed to predict the monitoring data under normal conditions. The results are illustrated in Figure 12.

**Figure 13:**
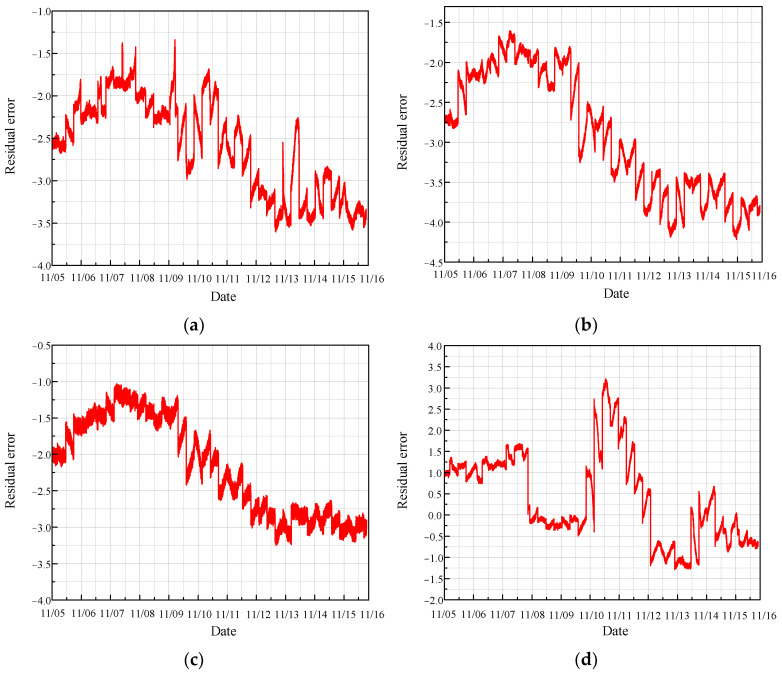
Residual error of normal prediction based on CNN: (**a**) results of sensor 102 MT_1_; (**b**) results of sensor 103 MT_1_; (**c**) results of sensor 104 MT_1_; (**d**) results of sensor 142 MT_1_.

Figure 13 presents the residual error between the predicted values and the true values under normal conditions, as obtained using the CNN model.

The prediction diagrams for each normal state demonstrate that the CNN model performs poorly, as the predicted values show significant deviation from the actual values. Furthermore, the residuals display irregular fluctuations, highlighting the model’s inadequacy in accurately reflecting the true behavior of the monitoring data. These findings indicate that the CNN model is not effective in reliably predicting the health status of the CRF pump.

(3) Deep AR

**Figure 14:**
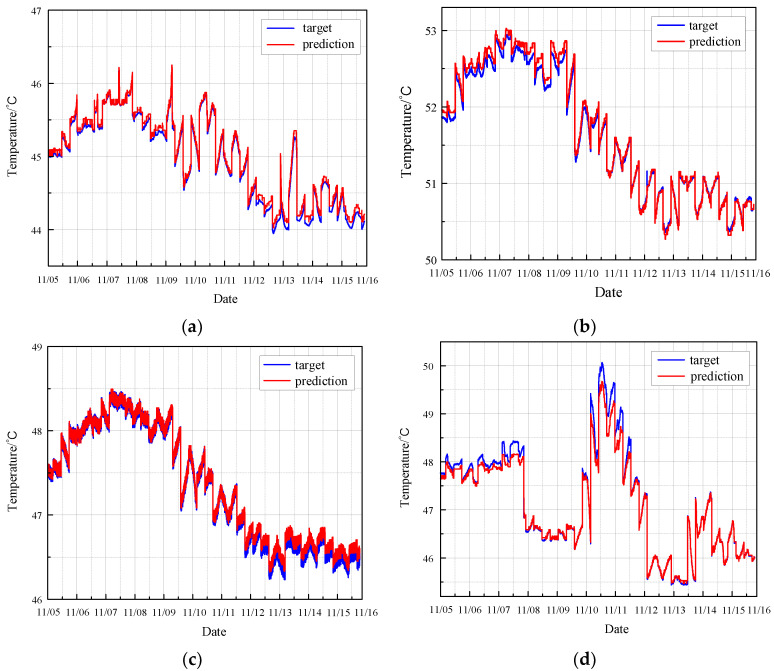
Prediction of normal condition based on Deep AR: (**a**) results of sensor 102 MT_1_; (**b**) results of sensor 103 MT_1_; (**c**) results of sensor 104 MT_1_; (**d**) results of sensor 142 MT_1_.

Applying the same rule, the Deep AR model is utilized to predict the monitoring data in a normal state. The outcomes of this prediction are shown in Figure 14.

**Figure 15:**
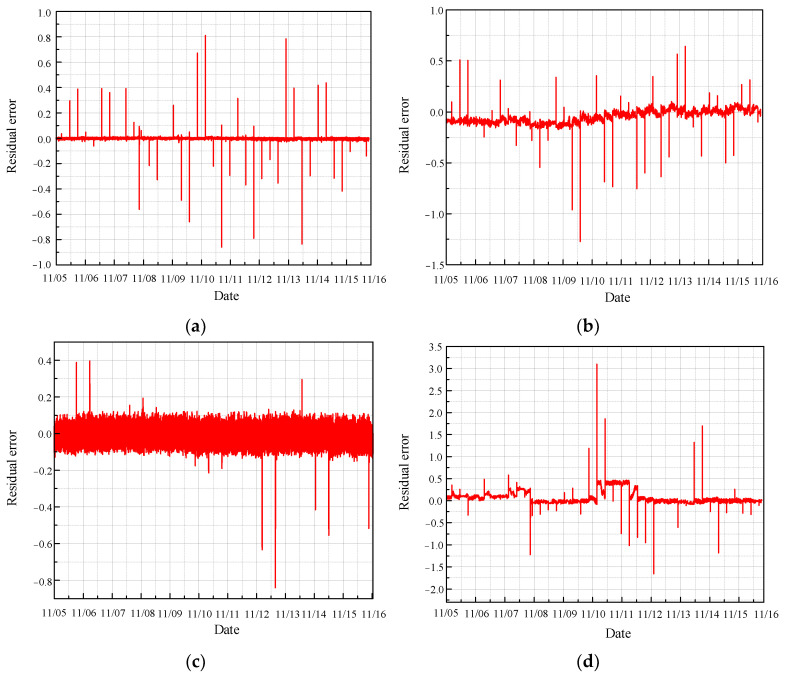
Residual error of normal prediction based on Deep AR: (**a**) results of sensor 102 MT_1_; (**b**) results of sensor 103 MT_1_; (**c**) results of sensor 104 MT_1_; (**d**) results of sensor 142 MT_1_.

The residual error between the predicted values and the true values under normal conditions, based on the Deep AR model, is illustrated in Figure 15.

Upon examining the normal conditions predicted by the Deep AR model, it is apparent that the predicted values align closely with the actual values, exhibiting only minor deviations in certain segments. The residual plot fluctuates around zero, indicating a general accuracy in the predictions. However, the magnitude of these fluctuations is more pronounced compared to those observed with the MGT model. This suggests that while Deep AR performs reasonably well in tracking the real values, it still exhibits higher variability in the residuals, reflecting areas where prediction accuracy could be improved.

(4) Temporal convolutional network

**Figure 16:**
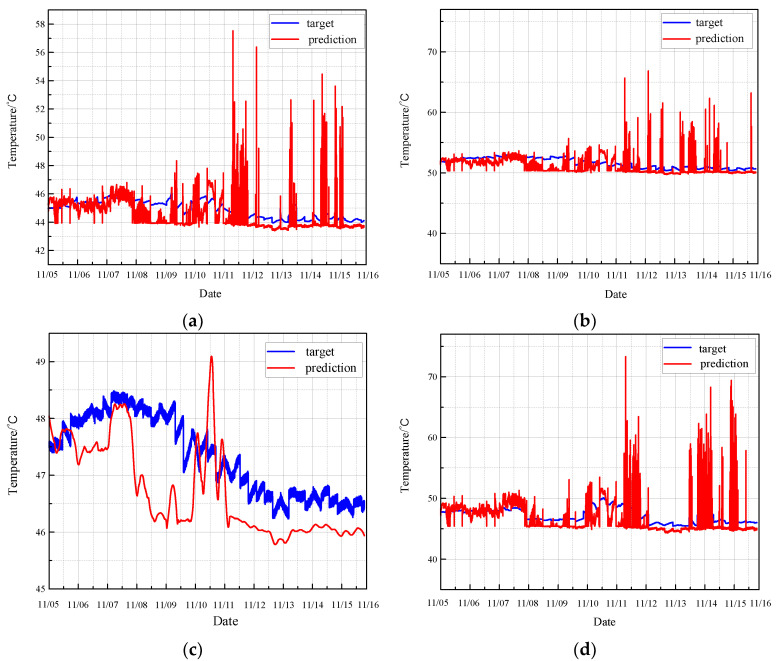
Prediction of normal condition based on TCN: (**a**) results of sensor 102 MT_1_; (**b**) results of sensor 103 MT_1_; (**c**) results of sensor 104 MT_1_; (**d**) results of sensor 142 MT_1_.

Applying the same rule, the TCN is used to predict the monitoring data under normal conditions. The results are displayed in Figure 16.

**Figure 17:**
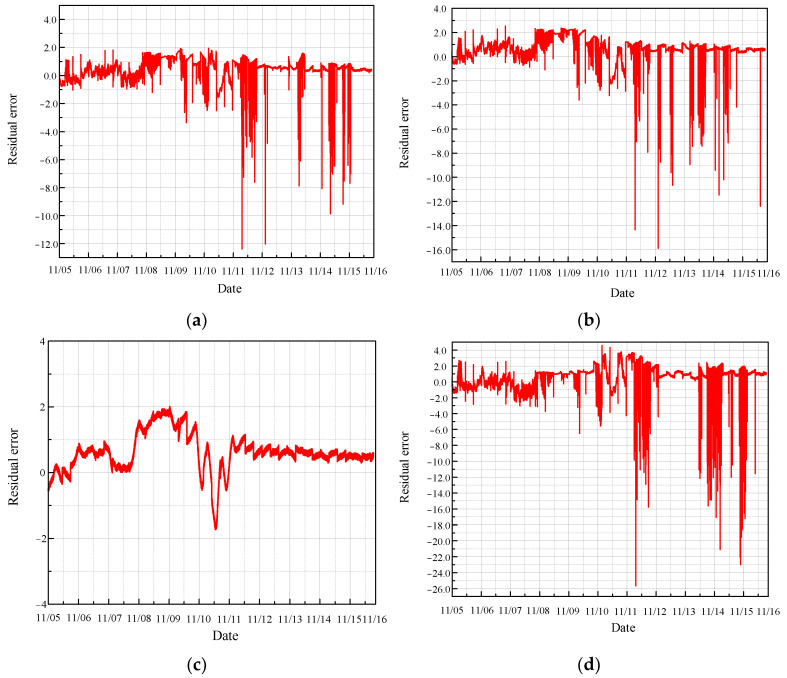
Residual error of normal prediction based on TCN: (**a**) results of sensor 102 MT_1_; (**b**) results of sensor 103 MT_1_; (**c**) results of sensor104 MT_1_; (**d**) results of sensor 142 MT_1_.

Figure 17 illustrates the residual error between the TCN model’s predicted values and the actual values under normal conditions.

The prediction map for normal conditions reveals that the TCN model performs inadequately. The predicted values exhibit substantial fluctuations and align with the actual trend only in limited sections. Moreover, the residual plot demonstrates significant variability, indicating a high degree of prediction inaccuracy. These results suggest that the TCN model struggles to reliably predict the health status of the CRF pump under normal operating conditions, as it fails to consistently capture the true behavior of the monitoring data.

The evaluation metrics for normal condition predictions, including MAE, MAPE, and RMSE, for the LSTM, CNN, TCN, Deep AR, and MGT models are summarized in Table 3.

Compared to the proposed MGT model, the LSTM, CNN, TCN, and Deep AR methods exhibit significant disadvantages in predicting the monitoring parameters of CRF pumps. This is because the multivariate time series composed of the CRF pump monitoring data involves complex interactions and dependencies among different time series. For instance, the value of one time series may be influenced by the current and historical values of other time series, and it may change in response to variations in those other time series. The aforementioned univariate time series prediction models are often unable to fully capture these interactions among multivariate time series, leading to poor prediction performance. This highlights the necessity of constructing graph data and processing it with a graph neural network when dealing with the various monitoring data of the CRF pump used in this study. Furthermore, it demonstrates that only the MGT model possesses the capability to construct an effective fault observer.

A comparative analysis of the training costs and generalizability of each model was conducted. The results are presented in Table 4 and Table 5, respectively.

From Table 4 and Table 5, it can be seen that some models may be more advantageous in terms of training time or resource usage; considering the overall performance of MGT, it demonstrates relatively lower training costs among all models. Moreover, MGT shows the lowest loss values on both validation and test sets, and the smallest performance difference between the training and validation sets, indicating its stronger generalizability.

#### 3.2.3. Construction of MGT Observer

**Figure 18:**
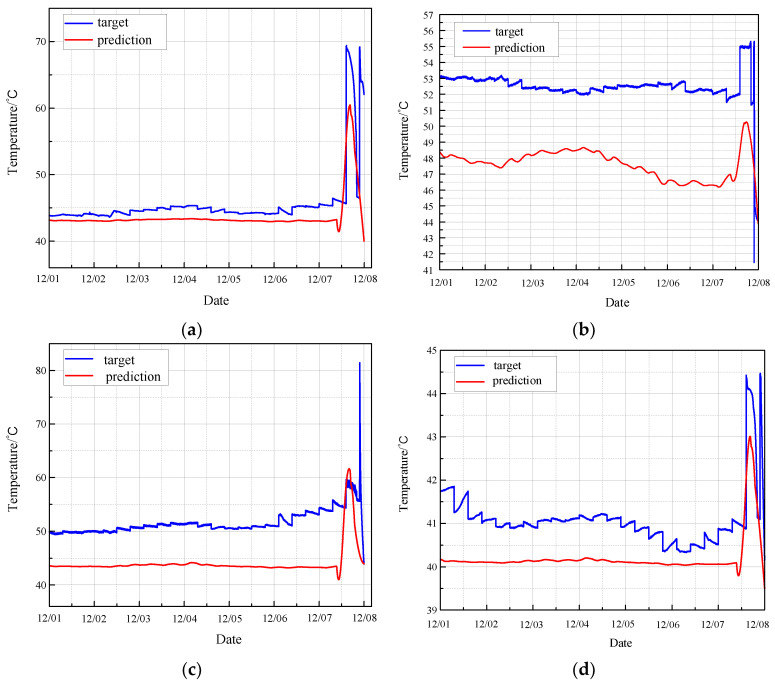
Fault observation based on MGT: (**a**) results of sensor 102 MT_1_; (**b**) results of sensor 103 MT_1_; (**c**) results of sensor 104 MT_1_; (**d**) results of sensor 142 MT_1_.

Based on the analysis presented in Section 3.2.1, a fault observer for the CRF pump is constructed using the MGT model. The fault occurred on 7 December, and the data from 1 December to 8 December was used to verify the effectiveness of the proposed fault observer. The monitoring data from the previously mentioned sensors was utilized to predict fault conditions, with the MGT model employed to analyze these data and forecast the fault stage. A detailed comparison between the predicted values and the actual monitoring data for the CRF pump during the fault period is illustrated in Figure 18.

**Figure 19:**
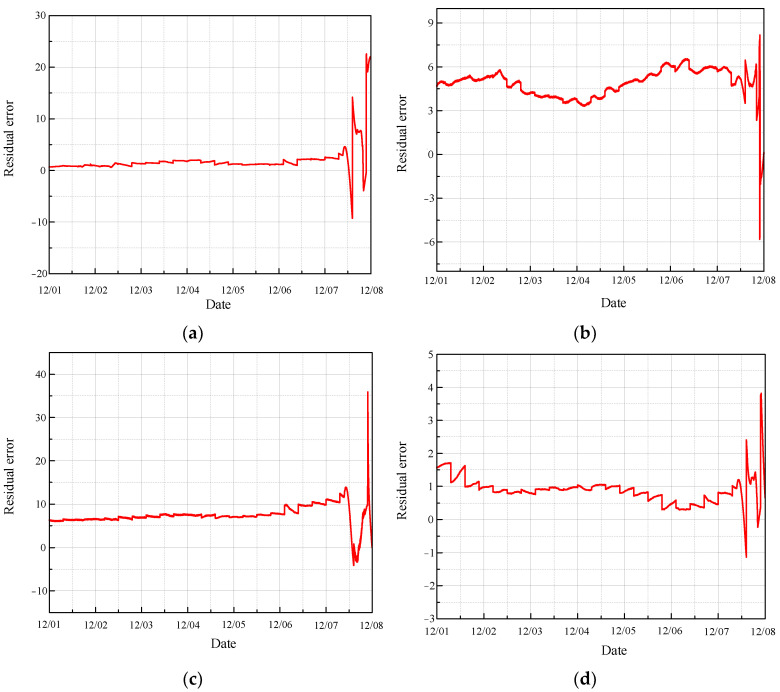
Residual error of fault observation based on MGT: (**a**) results of sensor 102 MT_1_; (**b**) results of sensor 103 MT_1_; (**c**) results of sensor 104 MT_1_; (**d**) results of sensor 142 MT_1_.

Using the same rule, Figure 19 depicts the residual error between the fault prediction values and the actual values based on MGT.

In each diagram, the fault prediction curve consistently lies below the actual value curve in the interval between the pre-fault and fault stages. This observation suggests that the fault prediction curve can serve as an effective threshold warning line. When the actual monitored values exceed this warning line, it indicates potential issues with the equipment that require immediate attention. By utilizing the fault prediction curve as a threshold, operators can identify early warning signs of equipment malfunction, thereby enabling timely maintenance and preventing further degradation of the CRF pump’s performance. This approach enhances the reliability and safety of the monitoring system, ensuring that potential faults are detected and addressed promptly.

The residual analysis of fault prediction reveals that the residual values significantly deviate from the stationary value of zero during the normal operation of the CRF pump. This deviation indicates the presence of faults or abnormal operations. The fault occurred on 7 December, and the results show that the fault observer detected the abnormality of equipment and issued an early warning as early as 1 December. This demonstrates that the proposed method can predict the abnormal condition of the CRF pump at least one week in advance. The ability to provide such early warnings highlights the effectiveness of the fault observer, allowing for timely interventions and maintenance to prevent further deterioration and potential failures, thereby enhancing the overall reliability and safety of the CRF pump system.

## 4. Conclusions

This paper presents a health monitoring model that uses temperature signals to predict the health states of CRF pumps. The temporal–spatial graph learning model, the MGT, is employed to forecast the trends of various monitoring data from CRF pumps. By utilizing the concept of a fault observer based on MGT, the model can provide early warnings of potential faults. The data collected from a CRF pump in a nuclear plant are used to validate the proposed model. The results demonstrate that the evaluation metrics for normal conditions—MAE, MAPE, and RMSE—are significantly improved, reducing to 0.5614, 1.2385, and 2.6554, respectively. These values are notably better than those obtained from the LSTM, CNN, TCN, and Deep AR methods. Additionally, the model proves capable of predicting fault conditions at least one week in advance, highlighting its effectiveness in early fault detection and prevention.

In future work, we will transform the proposed models into online learning modes, allowing health monitoring models to be dynamically adjusted based on real-time data for the online health monitoring of the CRF pump. Additionally, the scale of the graph data will be expanded, incorporating monitoring data from other components to construct a comprehensive graph structure of the entire equipment.

## Figures and Tables

**Table 1:** Detailed list of sensors.

No.	Code of Measuring Point	Location of Measuring Point	Unit
1	160 MT.H_1_	The output thrust bearing of the gearbox	°C
2	001 MI	The current of the motor	A
3	002 MI	The current of the motor	A
4	162 MT.H_2_	The top radial bearing of the motor	°C
5	164 MT.H_1_	The thrust bearing of the motor	°C
6	101 MT_1_	The upper radial bearing of the pump	°C
7	101 MT_2_	The upper radial bearing of the pump	°C
8	102 MT_1_	The upper radial bearing of the pump	°C
9	102 MT_2_	The upper radial bearing of the pump	°C
10	103 MT_1_	Pump thrust bearing	°C
11	103 MT_2_	Pump thrust bearing	°C
12	104 MT_1_	The thrust bearing of the pump	°C
13	104 MT_2_	The thrust bearing of the pump	°C
14	142 MT_1_	The lower radial bearing of the pump	°C
15	142 MT_2_	The lower radial bearing of the pump	°C

**Table 2:** Details of the data.

Data Type	Date of Data	Length of Data
Quantitative analysis data	5 October–16 November	460,482
Training data	5 October–19 October	222,000
Validation data	20 October–25 October	70,000
Normal test data	5 November–16 November	140,082
Fault test data	1 December–8 December	85,000

**Table 3:** Comparison of evaluation indexes.

	MAE	MAPE	RMSE
LSTM	14.4626	1.3612	27.7123
CNN	26.4723	3.9569	39.5432
TCN	21.6907	2.6734	30.5073
Deep AR	1.2864	1.9765	4.2572
MGT	0.5614	1.2385	2.6554

**Table 4:** Training cost of each model.

Model	GPU Type	CPU Cores	Training Time/Hours	Max Memory Usage/GB
LSTM	NVIDA GeForce RTX 4090	16	10	32
CNN			8	28
TCN			14	35
Deep AR			12	30
MGT			9	29

**Table 5:** Generalizability of each model.

Model	Validation Loss	Test Loss	Training Loss	Cross-Validation Mean	Cross-Validation Std Dev
LSTM	0.45	0.50	0.40	0.47	0.02
CNN	0.40	0.45	0.35	0.42	0.03
TCN	0.48	0.52	0.43	0.49	0.02
Deep AR	0.42	0.47	0.38	0.44	0.03
MGT	0.38	0.43	0.34	0.40	0.02

## Data Availability

Data underlying the results presented in this paper are not publicly available due to nuclear safety restrictions.
